# Chosen: A Life on the Margins of Care

**DOI:** 10.4269/ajtmh.25-0548

**Published:** 2025-11-04

**Authors:** Yvonne Karamagi, Edrin Jjuuko, Wandera Uthmaan Muluga

**Affiliations:** ^1^ Mildmay Uganda, Uganda;; ^2^ Mildmay Hospital, Uganda;; ^3^ Mildmay Research Centre, Uganda

I met her during a break. It wasn’t the usual calm of a late afternoon at Mildmay Hospital, but a quiet caused by events happening outside. It was the kind of silence that doesn’t come from taking a rest, but from something being suddenly broken. The kind of silence that happens after donors from far away ordered our work to stop.

She was seated alone on a wooden bench, elbows on knees, her file loosely tucked under one arm. She had not come for care that day. She had come because someone called her, someone who noticed her absence and reached for the gap. I was the doctor on duty, but it was not a day for duty. It was a day to quietly support others and make room for their emotions. Her name, here, is “Chosen.”

Chosen had lived with HIV since birth. Her parents, both gone before her first birthday, had left behind more than just their absence. They had passed on a condition, along with a lifelong connection to the health system that began long before she could understand what illness meant. By the age of six, she was already taking medicine every morning and every evening. She visited Mildmay frequently, where her blood was drawn, though no one explained the reason.

Her relatives had withheld the truth, perhaps out of protection or shame. So, she stopped taking the medication, while she was only six years old. Her body responded predictably. Her weight loss did not go unnoticed, and sadly, some in her community reduced her identity to her illness. Hurtful labels and whispers from classmates and neighbors deepened her sense of isolation. She remembered this with clarity, her voice flat, her eyes fixed on the clinic floor.

By the age of nine, she finally learned the truth about her illness. The way it was revealed, however, left her feeling hurt and unprepared, highlighting how crucial sensitive and supportive disclosure is for children. She was switched to second-line antiretroviral therapy, and with that transition came the first heavy realization that the path ahead was limited. At fifteen, the second-line regimen failed, and she was placed on third-line treatment. She referred to this regimen as the last chance.

Her treatment regimen was heavy for a child, sometimes more than two dozen small tablets a day. The side effects were difficult, and there were many days when she struggled through tears and nausea to keep going. She cried and vomited often. She carried her medicine packs to school in a stitched-up nylon pouch. On some mornings, she chose to skip school so she could come for her refill, walking more than three kilometres because her guardian had no transport fare. On other mornings, she came for her refills without having eaten. The pills sat heavy in an empty stomach, but she took them.

The Mildmay Hospital counsellors worked closely with her. Their voices soothed but also reoriented her reality. She came to accept her status and that acceptance brought change. She began to gain weight, her eyes brightened, her hair thickened, and she started telling jokes. Her medical record transformed, not in language, but in tone. From red notations of concern to green indicators of stability.

Then came January 2025.

The stop work order arrived as a memo. Programs funded by the U.S. President’s Emergency Plan for AIDS Relief (PEPFAR) were to pause operations. Staff were instructed to take leave and drug shipments were reportedly halted. The silence, once confined to the absence of disclosure in Chosen’s childhood, returned but this time across an entire health system.

For patients like Chosen, silence is fatal.

The third-line medications she needed are not part of standard public supply chains. They are expensive and scarce. The disruption in her view meant no guarantee of her next dose. No backup supply. No plan B. She stopped eating. She stopped speaking. In her despair, she began to consider ending her life. It was not a cry for pity, but a reflection of how deeply the system’s failure had pushed her into hopelessness.

When she told me this, her voice was calm. Her tone wasn’t seeking pity. It was simply factual. She had calculated the risks and to her, death by omission or by action were both plausible outcomes of the same systemic failure.

Her story was not unique. Others, too, were being negatively impacted. The clinic’s youth unit was paralyzed. Staff members gathered in emergency meetings, and we triaged patients based on regimen urgency and remaining stock. We asked ourselves how many doses we could stretch from reserve bottles.

In that moment, I did not feel like a doctor. I felt instead like a witness of system failure ahead.

We arranged for Chosen to receive psychological counselling. She sat with our lead counsellor who quietly handed her a two-month supply and reminded her of her strength. I saw Dr. Wandera that afternoon, his hands resting on a stack of patient record files. His silence was heavy. It held the weight of too many years working in vertical programs with shaky frameworks.

We knew how to provide care. What we lacked, suddenly and urgently, was power.

In Mildmay’s corridors, we began to speak differently. Our language shifted from “care plans” to “damage control.” From “adherence support” to “survival strategy.” From “scale-up” to “sustenance.” Each word marked the shrinking terrain of our clinical landscape.

What struck me most was how Chosen returned, not only to the clinic but to herself. After those few weeks, she resumed adherence. She began speaking to younger adolescents about disclosure and resilience. She reminded them that survival was not just biological. It was also political. It depended not just on swallowing pills, but on a world willing to keep those pills within reach.

She told one boy, not more than twelve, that he should think of his medicine as something he controls, not something that controls him. She smiled when she said it. It was a smile I had not seen before the disruption. I realized that, in her, the system had nearly failed, but not completely.

Sometimes, I wonder what would have happened had we not called her. Had that nurse not noticed her absence. Had the stock not been available. In global health work, the line between survival and loss is painfully thin, and that closeness remains a constant reminder of what is at stake. It follows clinicians through every partial refill and skipped dose. Chosen’s life continues not because the system worked but because people inside the system refused to let it fail completely.

To the global health community, to policymakers, to those who decide when the funds flow and when they freeze: we are asking you to see us. To remember that a stop work order is not just a bureaucratic pause, it is an act with consequences as real as missed heartbeats. To PEPFAR, whose support has saved millions of lives, we say thank you, but also: Your generosity saves lives. These fragile bodies need your helping hand. Adolescents like Chosen live on the edge of a system they did not build but depend on for every breath. They deserve uninterrupted care! Let us build a world where treatment is not a temporary privilege, but a durable promise.

Chosen reminds us that health systems built on foreign generosity carry within them a tension. They save lives, but they also suspend them. They empower, but they also decide who is counted. In this tension, clinicians do more than deliver care. We translate policies into survival. We hold hands while borders close around access. We sit with young people as they recalculate their chances.

What still stands out to me, even long after the stop work order ended, is this: Young people like Chosen are not just waiting for help. They act and keep things going, even in systems that often get disrupted. They carry more than just the burden of disease, they carry the hope and trust that we, as caregivers and decision-makers, won’t leave them behind when the funding changes.

It should never be that a child must choose between ending her life and uncertainty. Never that a youth must cry over transport fare instead of laughing in a classroom. Never that a clinic must ration medicine while donor negotiations unfold behind closed doors.

Her name is Chosen. Not because she was selected, but because she survived. Her story is not exceptional. It is exemplary. It is what happens when care persists despite abandonment. Let her life not be a testimony to luck, but to justice.

